# Impact of COVID-19 on mental healthcare of older adults: insights from Lebanon (Middle East)

**DOI:** 10.1017/S104161022000068X

**Published:** 2020-04-24

**Authors:** Rita Khoury, Georges Karam

**Affiliations:** 1 Department of Psychiatry and Clinical Psychology, Saint George Hospital University Medical Center, Beirut, Lebanon; 2 Faculty of Medicine, University of Balamand, Beirut, Lebanon; 3 Institute for Development Research Advocacy and Applied Care (IDRAAC), Beirut, Lebanon

The coronavirus disease 2019 (COVID-19) pandemic is causing a serious public health issue around the globe (Cascella, 2020). Its impact is even more devastating in low-to-middle income countries (LMIC) where healthcare systems risk to be overwhelmed very quickly (The Lancet, 2020). Older adults are at a higher risk of developing serious complications and death related to the virus, given their relatively weaker immune system and increased number of medical comorbidities compared to young adults (Sominsky *et al.*, 2020). Rigorous prevention measures such as social distancing and self-isolation undertaken by governments worldwide to curb the pandemic are being rigorously imposed on older adults. However, quarantine has been associated with a significant increase in depressive and anxiety symptoms in this population (Armitage and Nellums, 2020). Following the severe acute respiratory syndrome (SARS) epidemic in 2003, suicide rates in the elderly were shown to significantly increase in Hong Kong (Cheung *et al.*, 2008), hence the imminent need to promote mental well-being of older adults during this pandemic.

Lebanon is a small middle-income country in the Middle East located on the eastern coast of the Mediterranean. Its total surface area is around 10,452 km^2^ (Presidency of the Council of Ministers, 2020). Its population is estimated at around 7 million individuals (The World Bank, 2020), including 1.5 million Syrian refugees, the largest number of refugees per capita in the world, according to the latest reports from the United Nations High Commissioner for Refugees (UNHCR) (UNHCR Fact Sheet Lebanon, 2020). Lebanon currently reports the highest proportion of adults aged 65 years and above (10%) among all Arab countries (Central Administration of Statistics, 2012). This number is projected to increase to 21% by the year 2050 (Mehio *et al.*, 2015). Only 1.4% of the elderly population are institutionalized (Chemali *et al.*, 2008). The lifetime and 12-month prevalence rates of having a mental disorder in Lebanese older adults are 17.4% and 10.6%, respectively (Karam, 2016). Geriatric psychiatry is a burgeoning subspecialty in Lebanon, with currently only two geriatric psychiatrists (the authors RK and GK) registered at the Lebanese order of physicians practicing in an academic private university hospital in the capital, disserving the entire country. Primary care physicians and neurologists provide much of the mental healthcare for Lebanese older adults, because of the stigma related to mental illness, in addition to the scarcity of geriatric psychiatrists.

## Local challenges to geropsychiatry practice

Strategies to enhance mental well-being are important to put in place in a country with the highest proportion of older adults in the region. However, we are encountering several challenges. First, we noticed an initial decrease in older adults’ use of mental health services since lockdown was officially announced by authorities on March 15 (Lebanon-News, 2020). This phenomenon gradually improved with the patients adapting progressively to telepsychiatry services. From a therapeutic perspective, there has been a recent shift in the treatment modalities opted by older patients: due to contamination fear, most older patients and their caregivers have been avoiding inpatient treatment, even when needed. Also, electroconvulsive therapy, which is often indicated in this population for treatment-resistant or psychotic depression, was discontinued by most.

Counseling about lifestyle modifications (Khoury *et al.*, 2019) is being currently challenged by the confinement situation. Lebanese older adults’ routine normally involves taking care of grandchildren, helping their children with their daily chores, gathering socially over lunch every Sunday, and actively participating in religious services. All these activities have been withheld. Geriatric psychiatrists are now incited to find creative activities for their patients to remain physically, mentally, socially, and spiritually active while at home.

Dementia care has also been affected: patients and caregivers are no longer attending senior daycare program activities. Additionally, all therapeutic activities at the nursing homes have been discontinued; even visits by family members were banned.

Furthermore, there are rising concerns over an increase in elder abuse amid this pandemic, notably physical and psychological abuse by caregivers. Given the confinement, older adults have become more dependent on caregivers for providing them with food and medicine. Online orders and delivery of goods and medication to the doorstep are not readily available services in rural or underserved areas. Unfortunately, no televised awareness campaigns on elder abuse have been done to this date.

Finally, Lebanese older adults lose their public medical insurance after retirement, limiting their access to all medical services, at a time when they need it the most. Similarly, elder refugees lack appropriate resources (Chemali, 2018) to access mental health services during this crisis situation.

Additionally, research funds targeting the elderly and dementia have been reoriented toward COVID-19-related research.

## What are the solutions?

Considering the new realities imposed by this pandemic, several solutions are already being implemented and more are being considered. First, the practice of telepsychiatry has become very popular and is imposing itself as an effective and practical alternative to face-to-face psychiatric interviews even beyond the pandemic, given the scarcity of geriatric psychiatrists in Lebanon, and challenges related to transportation of frail, bedridden or institutionalized older adults. Although most older patients are showing initial resistance to change, they are progressively becoming accustomed to it, especially with the assistance of a caregiver. In the past few years, older Lebanese have increasingly become active users of social media and communication applications (especially WhatsApp). For the aim of staying connected with their loved ones within this pandemic, this phenomenon has further increased. For the convenience of older adults, most psychiatrists have been using the same applications they are familiar with to conduct psychiatric interviews rather than imposing on them novel technologies. However, telepsychiatry practice is not without any limitations, especially in specific populations such as the eldest old (aged 85 years and more) with a low level of education and those who live in remote areas with poor internet service. In these instances, regular phone consultations (on a fixed line) are being performed. Either way, psychiatrists have been recommending to their patients to keep socially connected using any available mean of communication to minimize the impact of social isolation shown to be the most significant predictor of developing mental disorders in Lebanese seniors (Karam, 2016). Additionally, we have been relying on the primary caregivers (family member or nurse at the nursing home) to describe abnormal movements, or to bring the camera closer to check for a patient’s tremor for instance, to assure a “virtual” physical exam.

Concerning caregivers, online support groups and webinars have been taking place on a weekly basis by academic centers and non-governmental organizations (NGOs): the Alzheimer Association Lebanon (AAL) has started weekly online meetings with caregivers to help them cope with issues related to dementia and confinement. Additionally, a hotline handled by a geriatric nurse at the AAL is provided to caregivers of patients with dementia to answer their urgent questions during this difficult period. Also, the Institute for Development, Research, Advocacy and Applied Care (IDRAAC) has been holding weekly public webinars about mental disorders potentially exacerbated by the COVID-19 pandemic. The attendance of these webinars and support groups has been increased by the fact that people are confined at home, raising awareness about mental illness on a large scale, which will most likely translate into better care of themselves and the older individuals they are taking care of. This unexpected positive outcome is encouraging these organizations to keep conducting online support groups and webinars on a regular basis, beyond the pandemic.

On a national level, actions taken by the ministry of public health to support vulnerable older adults financially and provide them with their basic needs, as well as ensure their legal protection from abuse are being promoted. We have also been advocating for reimbursement of telepsychiatry and other medical services to allow older adults to benefit from accessible, convenient yet affordable psychiatric assessments and treatments.

## Future directions

An important challenge to address in the future is setting the infrastructure for establishing advance directives among older Lebanese, in order to better preserve their autonomy should their decision-making capacity becomes altered by an acute serious illness. This pandemic shed light on the imminent need of the elderly to establish a living will as well as designate a legally authorized representative for medical-related decisions at an early age (<50 years old). Additionally, the British Medical Association discussed in its latest guidelines the issue of prioritizing mechanical ventilation for younger healthier patients over older patients with COVID-19 complications (British Medical Association, 2020), which application is raising ethical concerns around the globe, especially in LMIC countries where the number of ventilators can be limited. Although such a dilemma has not occurred yet in Lebanon, these recommendations incite our government and policy makers to revisit the construct of our ethical, social, and medico-legal systems. An ethical committee needs to be established to anticipate and address any future challenges related to sick older adults on a national level. Other future avenues involve developing and piloting technological applications that are elderly-friendly to fulfill their mental and social needs, as well as offer reliable information and tips in times of crisis. Mediterranean countries have recently shown a trend toward supporting innovative models of socio-ethical care, designed to benefit dependent older adults who lack standard social/family support: the European Union under the European Neighborhood Instrument, Cross-Border Cooperation, Mediterranean Sea Basin Program (ENI-CBC-MED) is currently funding a new project entitled “Development of a Transcultural social-ethical-care model for dependent population in Mediterra basin” (TEC-MED) (ENIC-CBC-MED, 2020). The aim of this project is to develop an innovative yet cost-effective socio-ethical care intervention framework which will adapt and improve social care policies in the aging population. A new organizational model involving public institutions and social care actors will be designed and validated in different Mediterranean countries, including Lebanon (under the direct supervision of author GK); the goal being to protect older adults from social isolation.

Finally, we learned from this pandemic that preparedness, flexibility, innovation, and a collaborative work between individuals, families, psychiatrists, NGOs, and governmental agencies are crucial to enhance the mental well-being of older adults and minimize the pandemic impact on our society.
